# Shining Light on Benthic Macroalgae: Mechanisms of Complementarity in Layered Macroalgal Assemblages

**DOI:** 10.1371/journal.pone.0114146

**Published:** 2014-12-01

**Authors:** Leigh W. Tait, Ian Hawes, David R. Schiel

**Affiliations:** 1 Marine Ecology Research Group, School of Biological Sciences, University of Canterbury, Christchurch, New Zealand; 2 National Institute of Water & Atmosphere, Riccarton, Christchurch, New Zealand; 3 Gateway Antarctica, University of Canterbury, Christchurch, New Zealand; College of Charleston, United States of America

## Abstract

Phototrophs underpin most ecosystem processes, but to do this they need sufficient light. This critical resource, however, is compromised along many marine shores by increased loads of sediments and nutrients from degraded inland habitats. Increased attenuation of total irradiance within coastal water columns due to turbidity is known to reduce species' depth limits and affect the taxonomic structure and architecture of algal-dominated assemblages, but virtually no attention has been paid to the potential for changes in spectral quality of light energy to impact production dynamics. Pioneering studies over 70 years ago showed how different pigmentation of red, green and brown algae affected absorption spectra, action spectra, and photosynthetic efficiency across the PAR (photosynthetically active radiation) spectrum. Little of this, however, has found its way into ecological syntheses of the impacts of optically active contaminants on coastal macroalgal communities. Here we test the ability of macroalgal assemblages composed of multiple functional groups (including representatives from the chlorophyta, rhodophyta and phaeophyta) to use the total light resource, including different light wavelengths and examine the effects of suspended sediments on the penetration and spectral quality of light in coastal waters. We show that assemblages composed of multiple functional groups are better able to use light throughout the PAR spectrum. Macroalgal assemblages with four sub-canopy species were between 50–75% more productive than assemblages with only one or two sub-canopy species. Furthermore, attenuation of the PAR spectrum showed both a loss of quanta and a shift in spectral distribution with depth across coastal waters of different clarity, with consequences to productivity dynamics of diverse layered assemblages. The processes of light complementarity may help provide a mechanistic understanding of how altered turbidity affects macroalgal assemblages in coastal waters, which are increasingly threatened by diminishing light quantity and altered spectral distributions through sedimentation and eutrophication.

## Introduction

The absorption of light by autotrophic communities and fixation of carbon crucially underpins energy transfer in almost all ecosystems. Yet, despite a rich history of photosynthetic research dealing with the spectral regions of photosynthetically active radiation (PAR) used by photosynthetic organisms [1]–[5], there have been few attempts to integrate measurements of spectral light attenuation and spectral light use by diverse assemblages of macroalgae. Biodiversity-ecosystem-function studies have usually found a positive relationship between plant diversity and primary productivity [6]–[10], but the ecophysiological mechanisms of complementary resource use have been poorly explored [11]. Although others have found that species identity [12] and total biomass [13]–[14] are important in accounting for variation in NPP, the high diversity and turnover in macroalgal assemblages inhabiting the sub-canopy beneath larger canopy forming macroalgae [15] suggests high niche differentiation. Pigment complementarity may, therefore, be capable of enhancing resource use efficiency within canopies, as has been demonstrated in phytoplankton assemblages [11], [16]–[17].

Macroalgal assemblages have been shown to outperform monocultures in rates of net primary productivity (NPP) [13], [18]–[19] and nutrient uptake [20], with assemblages able to make efficient use of the full daily range of irradiance with low energy spillage to light saturation even at midday [21]. The mechanisms by which NPP is enhanced within diverse assemblages are unclear, but niche complementarity has been shown to outweigh selection effects [22]–[25]. The niche complementarity hypothesis implies that plant species or functional groups occupy functionally distinct niches within an ecosystem and can therefore use resources more efficiently [6]–[9]. However, direct functional evidence for niche complementarity is scarce in the scientific literature [26], [27], an example being the demonstration of complementary use of nitrogen in macroalgal assemblages [20], [28]. Here we examine the potential for complementary light use in layered macroalgal assemblages and the mechanisms responsible for enhancing efficient resource capture and use.

Persistent losses of canopy-forming macroalgae represent long-lasting shifts in community structure [29]–[37], and can cascade to changes in primary productivity dynamics [38]–[41]. While a range of stressors can be responsible for the loss of canopy-forming algae, including nutrients [42], reduced light [31], herbivory [43] and rising temperature [44], the role of spectral changes to the light environment on diverse layered assemblages has been poorly explored. Given the importance of efficient resource capture in the functioning of whole macrophyte assemblages [45], [46], changes in the amount [47] and spectral distribution of light [48] caused by modification of nutrient and sediment regimes, may detrimentally affect the functioning and composition of benthic autotroph communities.

In phytoplankton communities, a high diversity of photosynthetic pigments is associated with elevated light capture and biomass [11], [16], [17], [49]. In contrast to terrestrial vascular plants, phytoplankton [50] and macroalgae [51] can contain a variety of photosynthetic pigments, resulting in a diverse array of colour variations within the photosynthetic thalli of rhodophyta, chlorophyta and phaeophyta. Thus, algal species may show large differences in their resource-use abilities which reflect the physiological traits underpinning any diversity–productivity relationship in assemblages. Further, within macroalgal assemblages, where light delivery to the lower portions of the canopy [52] and sub-canopy is limited [53], [54], there are large differences in photosynthetic properties between canopy and sub-canopy species (e.g., ratio of structural to photosynthetic tissues; [55]) and the thallus morphology of sub-canopy species can vary considerably from size and shapes of blades, filaments, or crusts, all with unique photosynthetic traits and patterns of self-shading. Combined with high concentrations of photosynthetic pigments in shaded portions of algal thalli [56], sub-canopy species are often efficient light harvesters, capable of driving total assemblage NPP above that of the canopy alone [21], [57].

Here we examine photosynthesis in layered macroalgal assemblages of varying species richness to identify the mechanisms of efficient light use and the potential for spectral niche complementarity. We manipulated species richness of the sub-canopy by randomly selecting species from the local species pool and assigning richness treatments of 4, 2 and 1 sub-canopy species of equal total biomass, at natural abundance. We also altered canopy density, again within natural ranges, for each level of richness to understand the interaction between degree of shading and sub-canopy species richness. Furthermore, the use of the PAR spectrum by assemblages of varying richness was tested at four different peak light wavelengths (460, 530, 585, 630 nm). The relative attenuation of downwelling PAR wavelengths was also measured, to determine the dominant wavelengths available for photosynthesis in clear and turbid coastal waters. We hypothesized that diverse macroalgal assemblages would be better able to make use of the entire PAR spectrum, whereas assemblages of lower diversity would either have lower rates of photosynthesis or show inefficient light use at specific wavebands within the PAR spectrum.

## Material and Methods

Macroalgae used for laboratory incubations were removed from Wairepo reef, Kaikoura, New Zealand (42° 25′ 13.45″ S, 173° 42′ 39.29″ E). The algal communities at this intertidal platform are dominated by *Hormosira banksii*, a canopy-forming fucoid alga found throughout New Zealand and south-eastern Australia that can extend to the shallow subtidal zone. Beneath the *H. banksii* canopy a range of perennial and ephemeral species are found throughout the year, with most of the diversity being in the sub-canopy (e.g., [58], [59]). Algal collections were done under the collecting permit of the School of Biological Sciences, Canterbury University from New Zealand's Ministry for Primary Industries.

Macroalgal samples were transported to the University of Canterbury, Christchurch, New Zealand in a chilled container and were transferred to a re-circulating seawater system under a 12∶12 light dark cycle for 24 hours before photo-respirometry incubations. All experiments were done within a temperature controlled room maintained at 15°C.

### Sub-canopy diversity, canopy density and assemblage NPP

Before assignment of diversity treatments, 6 quadrats were randomly placed in the mid-intertidal zone (50×50 cm) to identify the dominant species present in the sub-canopy layers of *H. banksii*-dominated assemblages at that time. In total, 23 macroalgal species were found during the surveys in July and August 2013. Of these, 10 species occurred consistently in the quadrats and had >5% cover in at least 1 quadrat were used in the experiments. These species were: greens, *Ulva sp.*, *Caulerpa brownii*; reds, *Lophothamnion hirtum*, *Hymenena palmata*, *Champia novae-zelandiae*, and *Polysiphonia strictissima*; browns, *Cystophora torulosa*, *Carpophyllum maschalocarpum*, *Adenocystis utricularis*, *Halopteris virgata*. Although the fucoids *Cystophora torulosa* and *Carpophyllum maschalocarpum* are canopy-forming algae in low-intertidal and subtidal zones, beneath the *H. banksii* canopy in the mid-intertidal zone, they are stunted and no greater than 10 cm tall. All species used to construct sub-canopy assemblages in the laboratory were therefore collected from beneath *H. banksii* canopies to ensure consistent photo-acclimation between species.

Calcareous algae, such as *Corallina officinalis*, are a dominant component of the cover of primary substrata beneath canopies within the intertidal zone of Southern New Zealand [60], occurring as low-lying turfs (1–2 cm high) or encrusting paints [58]. Although they are a ubiquitous component of macroalgal assemblages, corallines contribute little to total assemblage NPP [21], [40]. *Corallina officinalis* and other calcareous algae were omitted from photosynthetic performance experiments because of the accumulation of sediments and invertebrates within the turf and the necessity of collecting *C. officinalis* by chipping sections of sub-stratum, and its high mineral content, making it difficult to standardize biomass between treatments. *C. officinalis*.

In addition to the quadrat surveys, replicate plots of 25×25 cm (n = 5) were cleared for estimating the biomass of the *H. banksii* canopy and the total sub-canopy biomass (excluding corallines) to guide reconstruction during laboratory experiments. On average, the sub-canopy biomass was approximately 100 g wet weight per 625 cm^2^ plot (excluding corallines) and the *H. banksii* canopy biomass was approximately 500 g wet weight per 625 cm^2^ plot. Canopy density experiments were set up by adjusting natural densities to the area of incubation chambers (314 cm^2^). This equated to 350 g wet weight for a canopy cover of 100%, 233 g for 66% canopy and 117 g for 33% canopy. The sub-canopy species were scaled to 70 g wet weight per treatment.

Sub-canopy species composition was randomized among canopy density treatments, but kept consistent across each level of canopy density. Sub-canopy species combinations were randomly selected for three richness combinations; 4, 2 and 1 sub-canopy species (see Appendix S1 for species combinations). Each level of richness used six random combinations. Although testing all potential combinations would be ideal, we wanted to perform incubations for all richness and canopy-density combinations within a narrow time-frame to avoid photo-acclimation under prolonged laboratory conditions. Target biomass of the sub-canopy was always 70 g (wet weight ±5 g), so, for example, the treatment that contained 4 species had 17.5 g (±2 g) per species. After incubations, all algae were dried at 50°C for 24 hours and the dry weights used to standardize the photosynthetic data.

Macroalgal species were arrayed on a weighted gridded platform, to which thalli were fixed at the holdfast using plastic zip ties. Locations of macroalgae on the gridded platform were also randomised, but the canopy of *H. banksii* was centralised on the platform to properly simulate real *H. banksii* canopies (which show clumping of holdfasts). The platforms were then placed in incubation chambers where photosynthetic measurements were done.

### Photosynthetic measurements

The photosynthetic activity of macroalgal assemblages was tested using large (200 mm diameter, 300 mm high) photo-respirometry chambers, each with an internal circulating pump to ensure no boundary layers would form that could affect photosynthetic performance. We measured change in dissolved oxygen within the sealed photo-respirometers at irradiances of 0–2000 µmol photons m^−2^ s^−1^, which covered the full natural range, to generate photosynthesis-irradiance curves (*P–E*). Although emersed primary productivity can be an important component of total carbon fixation [38], our measurements focused on the dynamics of light-use and delivery within immersed assemblages. From these we were able to calculate photosynthetic parameters including respiration rates, photosynthetic efficiency (*α*), compensating irradiance, maximum primary productivity (*P*_m_), photoinhibition (*β*) and compensating irradiance (*E*_c_).

Algae were incubated under the various light intensities using Phillips Discharge metal halide lamps, limited to PAR wavelengths, with irradiance adjusted using neutral-density filters to give five light intensities (150, 600, 1150, 1750 and 2000 µmol photons m^−2^ s^−1^). NPP was estimated as rate of change in oxygen concentration over 20 minute periods, measured using a Hach LDO meter (Model HQ40d) incorporated into the chamber. Dark respiration was obtained by covering the chamber to omit light. Measurements of dark respiration were performed at least 30 minutes after algae had been exposed to light. In total, four replicate assemblages were incubated under each light intensity and canopy density combination. A full description of incubation protocols and the photo-respirometry apparatus can be found in Tait and Schiel 2010 [61].

### In situ spectral light attenuation

To obtain an initial assessment of light quality and quantity in coastal waters, the relative attenuation of downwelling PAR wavelengths *in situ* was characterised for comparison with the spectral regions of light used by macroalgal species and assemblages. The spectral characteristics of light were examined during summer 2013 (December 15–16) on the east coast of New Zealand's South Island (Banks Peninsula). In total, 6 spectral casts were done at 6 locations of high and low turbidity, to give relative measures under the same light conditions. Measurements were performed during two days with minimal cloud cover, following a period of relatively dry weather (i.e., low input of sediments and nutrients from terrigenous sources). These data represent a snapshot of the spectral characteristics of light attenuation in turbid vs. clear water, but it is important to note that there will be large seasonal variation in the attenuation of different PAR wavelengths due to variation in biological activity (e.g., phytoplankton abundance; [62], [63]) and environmental factors (e.g., rainfall, wave climate, tidal cycles; [47]).

Light measurements were done using a cosine Satlantic^©^ Multispectral Radiometer (OCR-507) with spectral channels set at 412, 470, 535, 565, 624, 670 nm. The spectroradiometer was lowered to depths of 0.1, 1, 2, 3, 4, 5, and 10 m, taking integrated measurements of light spectra and total downwelling irradiance at each depth and at three locations of high turbidity (near shore) and three locations of low turbidity (off-shore). Attenuation of downwelling light in waveband *λ* (*K*_d_(*λ*)), was calculated by log linear regression of irradiance against depth between 1 and 10 m [64]. The spectral attenuation of downwelling light at each of three offshore and three onshore sites were averaged, and used to compare relative spectral attenuation of downwelling light in turbid vs. clear water:
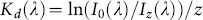



Where *I*_o_ and *I*_z_ are irradiance at the surface and depth z.

### Assemblage action spectra

Randomized species combinations, as in the canopy density experiment (Appendix S1), were used to test how relative photosynthesis across the PAR spectrum (i.e., action spectra) of assemblages is affected by sub-canopy diversity. We used coloured LED lights to illuminate macroalgal assemblages and measured oxygen change under four light colours (blue at 460 nm peak intensity, green at 530 nm, yellow at 585 nm and red at 630 nm). LED intensity varied between the colours so the number of LED units was adjusted to get even photon flux between colours (measured with a Skye PAR quantum light meter). Photon flux was set at 30 µmol m^−2^ s^−1^ (±3 µmol m^−2^ s^−1^) for all LED colours. While 30 µmol m^−2^ s^−1^ was low compared to the intensities reaching the canopy *in situ* [61], this is realistic for sub-canopy plants. Measurements of light (full PAR spectrum with Phillips Discharge metal halide lamps) transmittance through the canopy under laboratory conditions indicated that at an above canopy irradiance of 1500 µmol m^−2^ s^−1^, the sub-canopy irradiance was between 30–75 µmol m^−2^ s^−1^, depending on *H. banksii* canopy density. The spectral range of the LEDs was also examined using spectrometry (Ocean Optics USB2000) and was shown to have relatively narrow peaks (with a half-band width of 15–25 nm) and with <10% overlap between LED bands.

Action spectra were measured at 3 levels of species richness: 4, 2, and 1 sub-canopy species, each with and without an *H. banksii* canopy. The same randomized species combinations used for generating P–E curves were also used to generate action spectra (Appendix S1). Due to the low light intensity and the potential variation in respiration rates, gross primary productivity (GPP) was used to analyse differences in action spectra between richness treatments and canopy presence.

### Statistical analysis

To analyse differences between diversity and density treatments, *P–E* curves were fitted to test the differences in model parameters. The P–E curves were fitted using the equation:
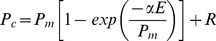
where *P*_c_ is the gross rate of photosynthesis, *P*_m_ is maximum NPP, *R* is the respiration rate, *α* is the photosynthetic efficiency and *E* is irradiance. The model parameters tested using F-tests were *P*_m_, *α* and *R*. Differences between canopy densities were analysed using these photosynthetic parameters (*P*_m_, *R* and *α*), as well as compensating irradiance (*E*_c_). Details on photosynthetic parameter calculation can be found in Walsby [65]. Furthermore, to estimate the relative contribution of the sub-canopy to assemblage primary productivity, NPP of the canopy-alone treatment was subtracted from the total assemblage treatments. The effects of canopy density, sub-canopy diversity and 4 light intensities (600, 1150, 1750, 2000 µmol m^−2^ s^−1^) on sub-canopy contribution were analysed using factorial ANOVA.

The effect of sub-canopy diversity with varying wavelengths was analysed with two-way ANOVA and Bonferroni post-tests. All data available online at DRYAD.

## Results

### Sub-canopy diversity and assemblage NPP

**Figure 1:**
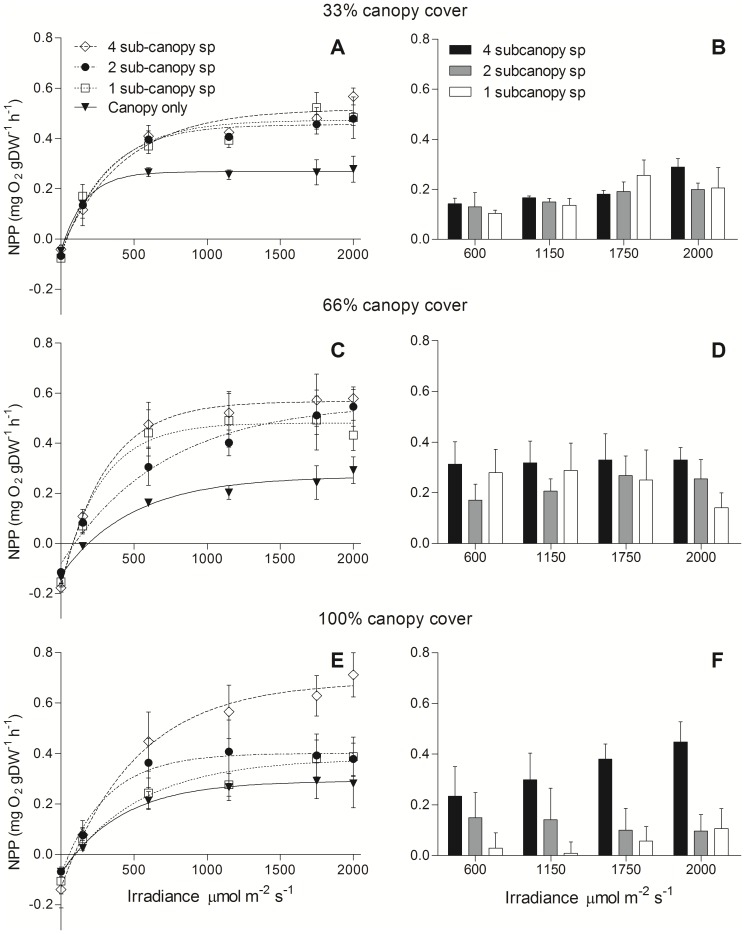
Net primary productivity (±SE) vs. irradiance (*P–E*) curves of assemblages with an overlying canopy of *H. banksii* at three densities and a sub-canopy layer composed of 1, 2 or 4 species (of equal total biomass). Figures show *P–E* curves for the three sub-canopy richness treatments and canopy alone (A, C and E), and the corresponding figures (B, D and F) show the NPP of the sub-canopy richness treatments with the NPP of the canopy subtracted. NPP is standardized by dry biomass of macroalgae to compare between canopy densities.

**Figure 2:**
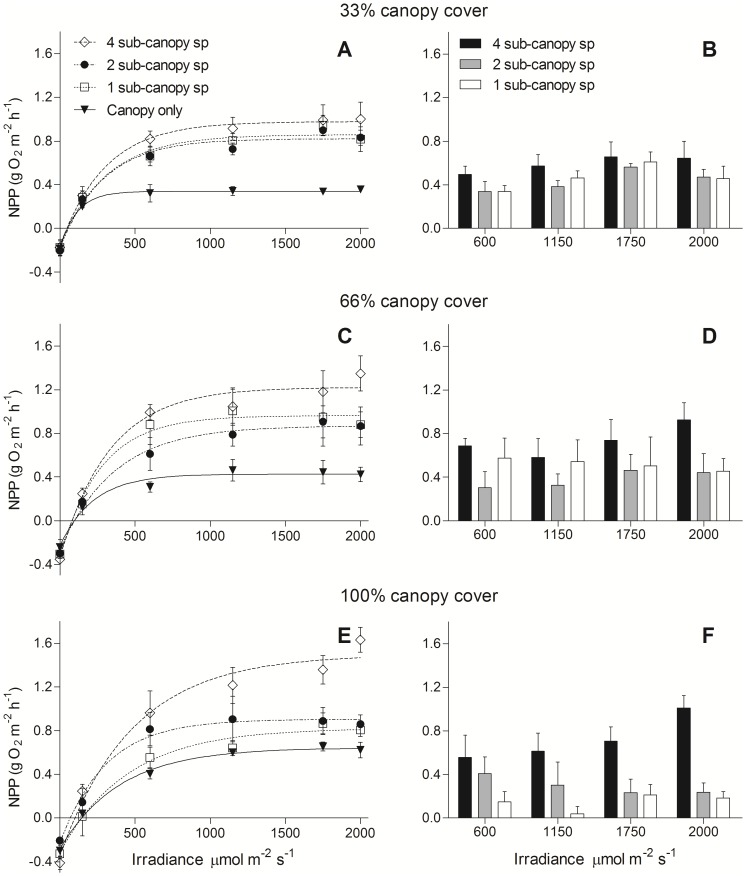
Net primary productivity (±SE) vs. irradiance (*P–E*) curves of assemblages with an overlying canopy of *H. banksii* at three densities and a sub-canopy layer composed of 1, 2 or 4 species (of equal total biomass). Figures show *P–E* curves for the three sub-canopy richness treatments and canopy alone (A, C and E), and the corresponding figures (B, D and F) show the NPP of the sub-canopy richness treatments with the NPP of the canopy subtracted. NPP is standardized by reef area to examine the effects of canopy density and sub-canopy richness on ecosystem function.

In all canopy-density treatments, average primary productivity increased from a minimum with the canopy alone, to a maximum where there were 4 species in the sub-canopy, regardless of whether the data were standardized by weight (Fig. 1 A, C, E) or by area (Fig. 2 A, C, E). There was, however, a significant effect of canopy density and sub-canopy diversity (diversity × density interaction, per gDW, F_4,108_ = 5.1, p = 0.001; per m^2^, F_4,108_ = 3.6, p = 0.009) on the sub-canopy contribution to NPP (i.e., Figs. 1 & 2 B, D, F). In the 100% canopy cover treatment, there was similar NPP in the treatments with the canopy alone, 1 sub-canopy, and 2 sub-canopy species, but the treatment with 4 species showed a 50–75% increase in NPP from 1200–2000 µmol m^−2^ s^−1^ compared to the next highest treatment NPP (Fig. 1 & 2 E). Where the canopy density was reduced to 33% and 66% cover, however, there was no difference between the three sub-canopy richness treatments, but at both lowered canopy densities, NPP was greater than with the canopy alone. Interestingly, the greatest NPP was seen in the full canopy treatment with highest sub-canopy richness (Fig. 1 & 2 E) suggesting synergistic light use at the high irradiance.

**Figure 3:**
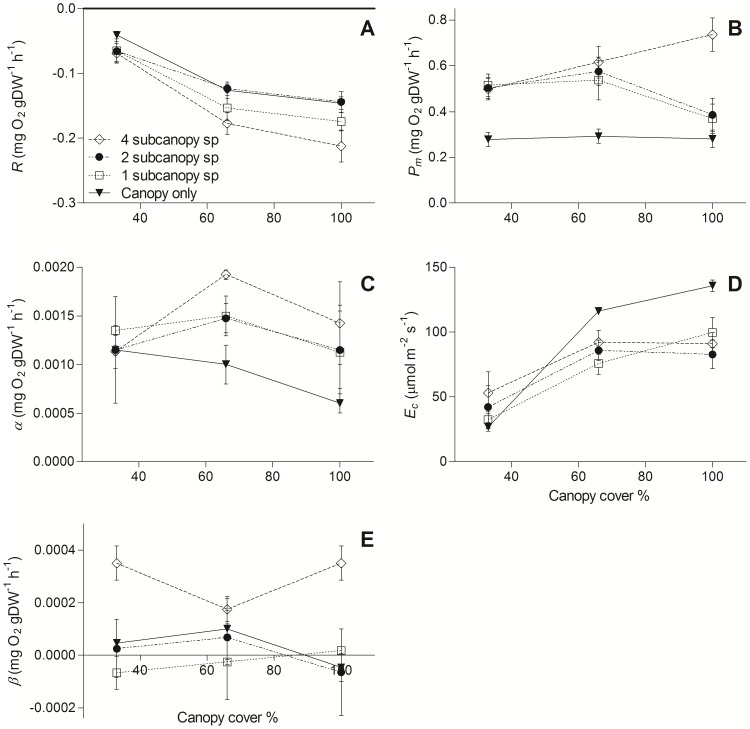
Photosynthetic parameters of layered assemblages of varying diversity and canopy density. Parameters shown are the respiration rate *R* (A), maximum NPP *P*_m_ (B), the photosynthetic efficiency *α* (C), compensating irradiance *E*_c_ (D), and photoinhibition *β* (E).

Photosynthetic parameters of layered assemblages (Fig. 3) were affected by canopy density (*R*, *E*_c_), sub-canopy species richness (*β*) or both (*P*_m_). *R* (Fig. 3 A) increased with canopy density and was highest in the 4 sub-canopy richness treatment at 100% canopy cover (F_3,60_ = 4.98, p = 0.0047). *P*_m_ (Fig. 3 B) varied between the sub-canopy richness treatments and canopy densities (richness × density interaction; F_6,60_ = 4.44, p = 0.0087) and the 4 sub-canopy species treatment had a consistently high rise in *P*_m_ across the canopy density gradient. Comparison of photosynthetic efficiency (*α*; Fig. 3 C) showed no trends between treatments or density (richness, F_3,60_ = 2.02, p = 0.13). *E*_c_ (Fig. 3 D) and tended to increase with canopy density, although in the 2 and 4 sub-canopy species treatments compensating irradiance stabilized between 66 and 100% canopy cover (richness × density interaction; F_6,60_ = 5.94, p = 0.002). *β* (Fig. 3 E) was positive (i.e., NPP increased at the highest irradiance) in the high sub-canopy richness treatment, but was negative (indicating photoinhibition) in the 1 and 2 sub-canopy species treatments at 100% canopy density (richness, F_3,60_ = 8.2, p = 0.00023).

Figure 3 conforms to the common practice of normalising NPP to biomass. Essentially the same pattern of response to canopy cover and sub-canopy species richness is observed when net photosynthesis is normalised to area (Fig. 2). In each treatment combination the presence of a sub-canopy increases the areal efficiency of light capture by photosynthesis, with the proportional increase tending to be greatest at 100% canopy cover and 4 sub-canopy species.

### Spectral light attenuation in coastal waters and assemblage action spectra

**Figure 4:**
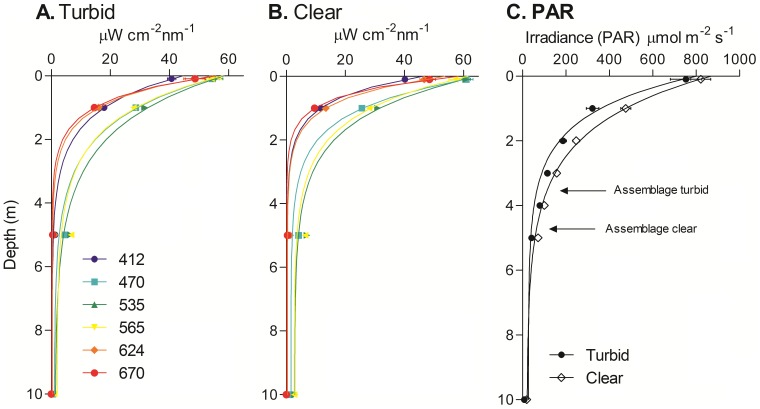
Light attenuation (±SE) with depth at six wavebands (412, 470, 535, 565, 624 and 670 nm) in turbid nearshore locations (A) and offshore locations (B). Graph C shows attenuation of total PAR for both clear and turbid water, with the estimated depth limits of *H. banksii* assemblages exposed to clear or turbid conditions (based on compensating irradiance). Compensating irradiance for *H. banksii* assemblages was 83 µmol m^−2^s^−1^ (Tait & Schiel 2011a).

Comparisons of light intensity within 10 metres of the surface between clear and turbid coastal waters showed variable attenuation across the PAR spectrum (Fig. 4). In turbid nearshore waters (Fig. 4A), all wavelengths were virtually undetectable by 10 m depth (yellow light, 565 nm had the highest intensity at 0.19 µW cm^−2^nm^−1^), but in clear offshore waters (Fig. 4B) most wavelengths were detectable at 10 m (green light, 535 nm had the highest intensity at 2.9 µW cm^−2^nm^−1^). The attenuation co-efficient for downwelling radiation in each waveband (*K*_d_ (*λ*)) was higher under turbid than clear conditions (Table 1), with violet (412 nm) and red (670 nm) wavebands showing the lowest proportional increase from clear to turbid conditions, and blue (470 nm), the greatest proportional increase. Spectral attenuation varied among turbid onshore and clear offshore sites (turbidity × wavelength interaction, F_5,72_ = 10.3, p<0.0001). Attenuation of total downwelling PAR (Fig. 4C) was affected by turbidity (depth × turbidity interaction, F_6,21_ = 3.3, p = 0.011). Estimated depth limits of *H. banksii* assemblages based on attenuation coefficients and compensation irradiance was reduced by approximately 1 m in turbid water.

**Table 1:** Attenuation co-efficient for downwelling radiation in each spectral waveband (*K*_d_ (*λ*) ± SE) in turbid, nearshore waters and clear offshore waters. Also shown is the ratio of Turbid: Clear.

	*K*_d_ (*λ*)					
Waveband	412 nm	470 nm	532 nm	565 nm	624 nm	670 nm
Clear	0.28 (0.019)	0.19 (0.016)	0.16 (0.025)	0.16 (0.025)	0.29 (0.024)	0.37 (0.01)
Turbid	0.56 (0.016)	0.35 (0.015)	0.29 (0.014)	0.28 (0.015)	0.45 (0.037)	0.59 (0.02)
Ratio	2.0	1.8	1.8	1.7	1.6	1.6

**Figure 5:**
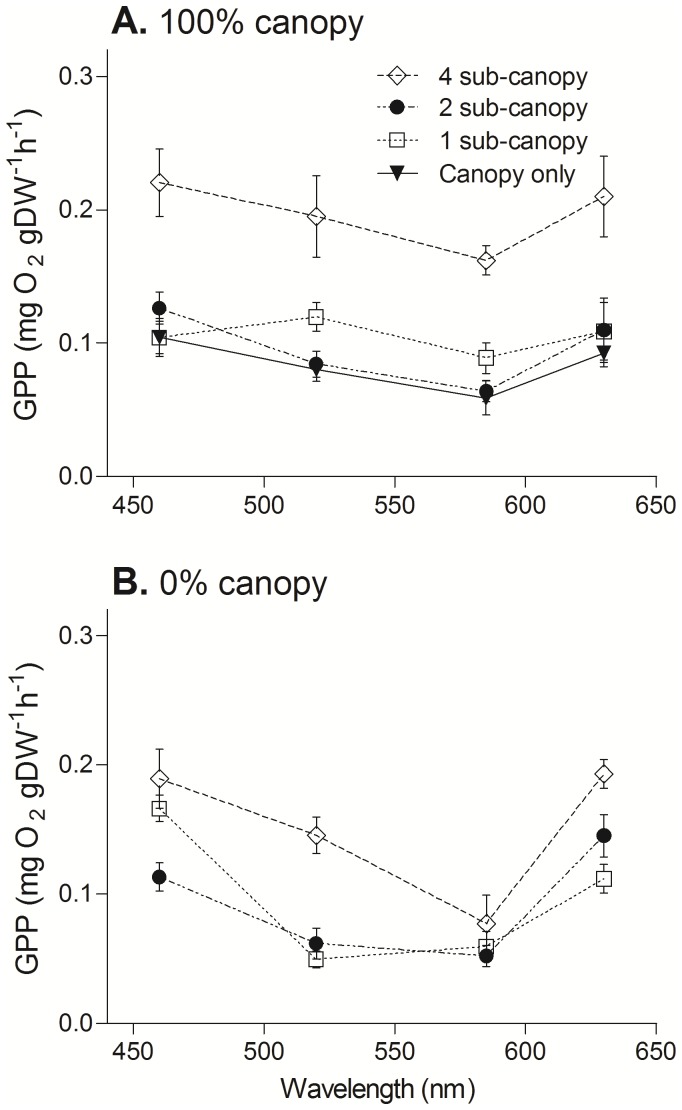
Action spectra of macroalgal assemblages of varying diversity, with (A) and without (B) an overlying *H. banksii* canopy. Action spectra show the rate of GPP at four peak wavelengths (colours) for two canopy treatments (0% and 100% canopy cover) and three diversity treatments (4, 2 and 1 sub-canopy species). Action spectra of the *H. banksii* canopy (solid line) are shown for comparison. Data are standardized to grams dry weight of algal material.

Photosynthetic action spectra of assemblages with an overlying *H. banksii* canopy showed relatively even use of the light spectrum, and species-poor treatments (1 or 2 sub-canopy species) had much lower gross primary productivity (GPP) than the treatment with 4 sub-canopy species (Fig. 5A). In the absence of the *H. banksii* canopy, however, action spectra of the sub-canopy were more variable, but the species-rich treatments had the highest GPP (Fig. 5B) and blue and red light showed slightly higher GPP at most levels of richness (F_3,60_ = 3.5, p = 0.022). Without the *H. banksii* canopy the 4 sub-canopy species treatment had the highest rates of GPP, but the 1 and 2 sub-canopy species treatments were much more variable across the light spectrum (richness × wavelength interaction, F_6,60_ = 3.2, p = 0.013). Overall, there was a significant interaction between Canopy and Treatment; in particular, the most diverse assemblage had the greatest GPP, and this occurred under the *H. banksii* canopy. Outside of the canopy, there was clearly differential use of light spectra, with diverse assemblage able to use all four light wavelengths more efficiently than assemblages with 1 or 2 sub-canopy species.

Assemblages at most levels of species richness with and without canopies had slightly higher GPP at blue (c. 460 nm) and red (c. 630 nm) wavelengths (Fig. 5). The preferential increases in attenuation coefficients for 6 wavebands within the PAR region showed differentially greater increases in attenuation of blue than red light in turbid conditions compared to clear conditions (Table 1), with implications for the photosynthetic performance of diverse macroalgal assemblages under high sediment loads.

## Discussion

Complementary light use has been suggested as one possible mechanism yielding positive effects of species diversity on ecosystem function [6], [11], [66], [67], but few studies have attempted to explore this [26], [27]. In this study we show that within macroalgal assemblages, sub-canopy species richness enhances primary productivity, both when normalised to dry weight and to area. High canopy densities enhanced the role of sub-canopy diversity, suggesting that under highly shaded conditions diversity of photosynthetic pigments or diverse thallus morphologies increases resource use efficiency. Elevated NPP per unit biomass in the presence of many species of understory macroalgae may in part be due to the reduced non-photosynthetic biomass in these taxa relative to canopy species, but increased efficiency per unit area shows that species-rich assemblages enhance the overall conversion of solar energy to photochemical energy. We also show that the partitioning of the light spectra between species with variable pigment compositions may be the mechanism through which assemblages are able to enhance rates of NPP, similar to enhanced nitrogen use in diverse macroalgal assemblages [20].

Variation in action spectra has rarely been considered as a mechanism for the enhancement of ecosystem function with diversity. However, there is evidence to suggest that inter-species variation in spectral absorption may be a vital mechanism of resource use complementarity, as has been found for phototrophic microorganisms [11], [16]. Although there have been studies detailing the variation in absorption spectra between macroalgae [1]–[5], [51], the integration of multiple action spectra within macroalgal assemblages caused a smoothing of the action spectra, as indicated by the enhanced use of green-yellow wavelengths in species-rich assemblages and higher overall GPP compared to species-poor assemblages. Our research provides the first evidence of the potential for spectral niche complementary in macroalgal assemblages and highlights the necessity of considering the canopy layering of vegetation stands to understand the physiological consequences of environmental stressors.

Diverse natural sub-canopy assemblages of macroalgae have been shown to compensate for the loss of canopy-forming species [57] and the presence of multiple functional groups may be critical to the maximal use of light and buffering the effects of canopy disturbance [40]. Variation in peak wavelength absorption and action spectra between species could be responsible for the observed increases in primary production in assemblages/polycultures compared to monocultures [12], [13], [20] and we show that structured macroalgal stands, including canopy species, help to enhance total light capture and hence NPP. The species-specific differences in pigment composition responsible for enhanced primary productivity in diverse assemblages may represent an evolutionary response to the low light environment beneath macroalgal canopies, maximising total light capture and potentially exploiting alterations to the light spectrum caused by the canopies themselves. To our knowledge, such effects are yet to be explored, but spectral light quality has been shown to change when attenuated through water columns containing phytoplankton [62] and some types of sediments [63]. Although the intertidal zone may receive very high light intensities relative to sub-tidal benthic reefs, shading by canopy-forming algae may produce a highly competitive sub-canopy assemblage, which in these systems have high levels of species richness and a high turnover of species through time [60].

Spectral attenuation of light has been well-characterised in coastal and oceanic waters [63] and has led to some major advances in our understanding of marine systems, including the development of satellite models of global phytoplankton NPP [68], [69]. Here we show variable attenuation of PAR wavelengths in turbid and clear water, as well as potentially decreasing depth ranges for macroalgal assemblages in turbid waters, as determined by the compensating irradiance of *H. banksii* assemblages [21]. In our study, the compensation depth in slightly turbid waters was 1 m less than that in clear waters. Such shallowing of depth limits has been well characterised in some regions including the Baltic Sea [70], southern Australia [31], and the Mediterranean [29], but the consequences of these shifts in depth profiles to whole assemblage primary productivity requires greater attention. Human-mediated alteration of sediment and nutrient regimes has the potential to alter both the quantity [47] and quality (spectral light distribution; [63]) of light reaching the benthos, with consequences to the operation of complementary light use, and the biodiversity of sub-canopy assemblages.

Loss of canopy-forming macroalgae and the proliferation of turf-forming algae in degraded near-shore habitats worldwide have largely been attributed to changes in nutrient and sediment regimes [31], [34], [35], [37]. Inherent differences in light-use efficiency of sub-canopy species [55] may be responsible for shifting species composition under increasingly turbid conditions due to higher pigment concentrations and reduced investment in structural tissues of sub-canopy species relative to canopy-forming species. While we show that species-rich macroalgal assemblages are able to maximise resource capture through variable action spectra and thallus morphologies, the consequences of a degraded light environment on the structure and composition of macroalgal assemblages is poorly understood. However, changes in the spectral light environment caused by increased sediment run-off and eutrophication [47], [63] may benefit some species over others. In particular removal of specific wavelengths through the water column (e.g., blue and red light absorbed by phytoplankton) may promote the growth of macroalgal species able to make use of yellow and green wavelengths (i.e., red algae [1]). Understanding the photo-physiological characteristics of canopy and sub-canopy species is likely to shed some light on the consequences of altered light attenuation on primary production dynamics and community composition.

## Supporting Information

Appendix S1 **Experimental combinations of sub-canopy species used at three levels of species richness.**
(DOCX)
