# Supplementary material for: Shining Light on Benthic Macroalgae: Mechanisms of Complementarity in Layered Macroalgal Assemblages
Source: PLoS One. 2014 Dec 1;9(12):e114146. doi: 10.1371/journal.pone.0114146 (PMC4250189; doi:10.1371/journal.pone.0114146)
Supplement: Appendix S1 — Experimental combinations of sub-canopy species used at three levels of species richness. (DOCX) [file pone.0114146.s001.docx]

Appendix S1. Experimental combinations of sub-canopy species used at three levels of species richness.

| 1 Species | 2 Species | | 4 Species | |
| --- | --- | --- | --- | --- |
| *Cystophora torulosa* | *Champia noveauzealandia, Cystophora torulosa* | *Ulva sp, Lophothamnion hirtum* | *Cystophora torulosa, Halopteris virgata, Champia noveauzealandia, Hymenena palmata* | *Lophothamnion hirtum, Champia noveauzealandia, Adenocystis utricularis, Caulerpa brownii* |
| *Carpophyllum maschalocarpum* |  |  |  |  |
| *Hymenena palmata* | *Hymenena palmata, Halopteris virgata* | *Caulerpa brownii, Adenocystis utricularis* |  |  |
| *Lophothamnion hirtum* |  |  |  |  |
| *Caulerpa brownii* | *Cystophora torulosa, Carpophyllum maschalocarpum* | *Champia noveauzealandia, Polysiphonia strictissima* | *Adenocystis utricularis, Halopteris virgata, Caulerpa brownii, Carpophyllum maschalocarpum* | *Carpophyllum maschalocarpum, Ulva sp., Polysiphonia strictissima, Lophothamnion hirtum* |
| *Ulva sp.* |  |  |  |  |
|  |  |  |  |  |
|  |  |  |  |  |
|  |  |  | *Ulva sp., Cystophora torulosa, Hymenena palmata, Champia noveauzealandia* | *Cystophora torulosa, Halopteris virgata, Polysiphonia strictissima, Carpophyllum maschalocarpum* |
|  |  |  |  |  |
|  |  |  |  |  |
|  |  |  |  |  |
